# Evaluation of Anesthesia Profile in Pediatric Patients after Inguinal Hernia Repair with Caudal Block or Local Wound Infiltration

**DOI:** 10.3889/oamjms.2016.023

**Published:** 2016-02-03

**Authors:** Aleksandra Gavrilovska-Brzanov, Biljana Kuzmanovska, Andrijan Kartalov, Ljupco Donev, Albert Lleshi, Marija Jovanovski-Srceva, Tatjana Spirovska, Nikola Brzanov, Risto Simeonov

**Affiliations:** 1 *University Clinic of Surgery, KARIL, Faculty of Medicine, Ss Cyril and Methodius University of Skopje, Skopje, Republic of Macedonia*; 2 *University Clinic of Surgery, ER Department, Faculty of Medicine, Ss Cyril and Methodius University of Skopje, Skopje, Republic of Macedonia*; 3 *University Clinic for Pediatric Surgery, Faculty of Medicine, Ss Cyril and Methodius University of Skopje, Skopje, Republic of Macedonia*

**Keywords:** Caudal block, Local wound infiltration, Pediatric analgesia, Hernia Inguinalis, Buivacaine

## Abstract

**AIM:**

The aim of this study is to evaluate anesthesia and recovery profile in pediatric patients after inguinal hernia repair with caudal block or local wound infiltration.

**MATERIAL AND METHODS:**

In this prospective interventional clinical study, the anesthesia and recovery profile was assessed in sixty pediatric patients undergoing inguinal hernia repair. Enrolled children were randomly assigned to either Group Caudal or Group Local infiltration. For caudal blocks, Caudal Group received 1 ml/kg of 0.25% bupivacaine; Local Infiltration Group received 0.2 ml/kg 0.25% bupivacaine. Investigator who was blinded to group allocation provided postoperative care and assessments. Postoperative pain was assessed. Motor functions and sedation were assessed as well.

**RESULTS:**

The two groups did not differ in terms of patient characteristic data and surgical profiles and there weren’t any hemodynamic changes between groups. Regarding the difference between groups for analgesic requirement there were two major points - on one hand it was statistically significant p < 0.05 whereas on the other hand time to first analgesic administration was not statistically significant p = 0.40. There were significant differences in the incidence of adverse effects in caudal and local group including: vomiting, delirium and urinary retention.

**CONCLUSIONS:**

Between children undergoing inguinal hernia repair, local wound infiltration insures safety and satisfactory analgesia for surgery. Compared to caudal block it is not overwhelming. Caudal block provides longer analgesia, however complications are rather common.

## Introduction

Prophylactic analgesia with local anesthetics is an attractive concept, especially in pediatric practice, because the evaluation of pain can be very challenging in young children [1]. In contrast to opioids, local anesthetics can be administered safely, and in recent guidelines regional anesthesia is accepted as the cornerstone of post-operative pain relief in the pediatric patients [2]. Although regional anesthesia holds a good safety record overall [3], the global experience with pediatric regional anesthesia is still quite low; even the most commonly performed procedure, caudal block, represents only 2.5% of all central neuraxial blocks performed [4]. Determining the risk-benefit ratio is rather difficult for techniques that are relatively rarely performed. Wound infiltration can produce reliable analgesia for superficial skin surgery. Infiltration itself is extensively used by pediatricians, surgeons and emergency physicians for skin laceration repair or minor superficial surgery [1]. Several studies have compared the local anesthesia so far, including: ilioinguinal and iliohypogastric nerve block plus subcutaneous injection by the surgeon against the caudal anesthesia [5-7]. But, to our knowledge there is still no study comparing the local wound infiltration by itself and caudal anesthesia. The aim of this study was to evaluate anesthesia and recovery profile in pediatric patients after inguinal hernia repair with caudal block (CB) or local wound infiltration (LWI).

## Methods

The study was approved by the Institutional Review Board of and was registered at Clinical Trials.gov (registration number: NCT02620566). This randomized double-blinded study was conducted at a single tertiary medical centre (“Mother Teresa”) in Skopje, Republic of Macedonia between September and December 2015. After obtaining written information consent from parents, we enrolled a total of 80 children aged 6 months to 7 years of ASA physical status I or II, undergoing unilateral hernia repair. Exclusion criteria included a history of developmental delay or mental retardation, type I diabetes, known or suspected coagulopathy, known allergy to any local anesthetic, known congenital anomaly of the spine, or signs of spinal anomaly or infection at the sacral or inguinal region.

For all the patients included in this trial (n = 80), a standardized anesthetic protocol was used. No premedication was administered. Anesthesia was induced with 2–3 mg/kg of propofol or 8% of sevoflurane in 100% oxygen. Standard monitors including: electrocardiography, noninvasive arterial pressure, pulse oximetry, carbon dioxide, and gas analyzer were applied during induction and maintenance of anesthesia. The airway was established by using a laryngeal mask airway (LMA). Anesthesia was maintained with sevoflurane, and depth of anesthesia was adjusted accordingly with a goal of 80–120% baseline arterial pressure and 4.7–6 kPa end-tidal carbon dioxide (EtCO_2_). Spontaneous breathing was maintained during surgery. After completion of surgery, the LMA was removed, and the child was sent to a post-anesthetic care unit (PACU) so long as there was no compromise in airway or hemodynamic instability per operatively.

According to the analgesic method used, all patients were allocated to two groups. Group C (caudal) (n = 40) and Group L (local wound infiltration) (n = 40). Enrolled children were randomly assigned to either Group C or Group L according to a computer-generated randomization table. For caudal blocks, Group C received 1 ml/kg of 0.25% bupivacaine (maximum volume 20 ml); Group L received 0.2 ml/kg 0.25% bupivacaine (maximum volume 4 ml) applied as local wound infiltration. An investigator who did not participate in the care of the enrolled children prepared all study medications according to group assignment. Another investigator, performed local infiltration or caudal blocks in all patients.

After induction of anesthesia, children from Group C were placed in a left lateral decubitus position. After the sacral cornua and hiatus were identified, the location of needle entry site was marked. Then a 5 cm short beveled 22 G block needle was inserted into the sacral epidural space. An aspiration test was conducted to exclude intravascular placement before injection administration.

The patients allocated in Group L received local infiltration of the surgical area with 0, 25% bupivacaine 10 min before skin incision. The infiltration technique was standardized as follows: the subcutaneous tissue in the proposed area was infiltrated with 0.25% bupivacaine before incision. A 16-mm 26-gauge needle was inserted in the center of the area, and the medial and lateral parts of the proposed skin incision were infiltrated. With the needle still in central position, a fan-shaped application was administered under the external abdominal fascia.

Surgery was initiated ten minutes after performing the caudal block or local infiltration. The caudal block or local infiltration is considered to have failed if the patient moved his or her limbs, had an increased heart rate, had an increase in mean arterial pressure, or both of more than 15% compared with baseline during the surgery. In such instances, the patient is to be withdrawn from the study and treated with 1–2 μg/kg of fentanyl.

Another investigator who was blinded to group allocation provided postoperative care and assessments. Postoperative pain was assessed using the Children’s Hospital of Eastern Ontario Pain Scale (CHEOPS, 0–10) [8] and the Faces Legs Activity Cry Consolability tool (FLACC, 0– 10) [9] at 15 min, 30 min and 1, 2, and 3 h after operation. A child with a score of more than 4 on both CHEOPS and FLACC received 0.5 μg/kg of fentanyl i.v. for rescue analgesia.

Motor function was assessed using the following scale: 0, no motor block; 1, able to move legs; 2, unable to move legs. Assessment of sedation was done with objective score based on eye opening: 0- spontaneously, 1- on verbal stimulation, 2- on physical stimulation. The presence of other adverse events was evaluated as well including: bradycardia, hypotension, respiratory depression, wound infection, fever, wound dehiscence, retching, vomiting, agitation, or urinary catheterization. Hypotension and respiratory depression were defined as 80% of baseline arterial pressure and ≤ 95% of pulse oxygen saturation, respectively. The decision to place a urinary catheter for urinary retention and the evaluation of micturition were made by an urologist. Analgesia on ward was provided with oral acetaminophen (15 mg/kg). The time for first supplemental oral acetaminophen demand (first acetaminophen time) was defined as the time from the end of surgery to the first registration of more than 4 on both CHEOPS and FLACC by the investigator.

Twenty-four hours after surgery, reports on delayed side-effects and demands for rescue acetaminophen from the child were gathered. The investigator, who was blinded to the treatment group, documented these data with the medical records. Children were discharged from the hospital after 24 h if they met the following discharge criteria: conscious, hemodynamically stable, tolerating oral intake, voiding, walking in an appropriate manner for age, with the absence of retching, vomiting, and other side-effects [10].

Statistical analysis Statistical analysis was performed by using SPSS 17.0. Data were expressed as mean and ± standard deviation and statistically analyzed using Student’s t-test, the Mann–Whitney rank-sum test and the test of Difference. A value less than 0.05 were considered as statistically significant for all tests.

## Results

**Figure 1:**
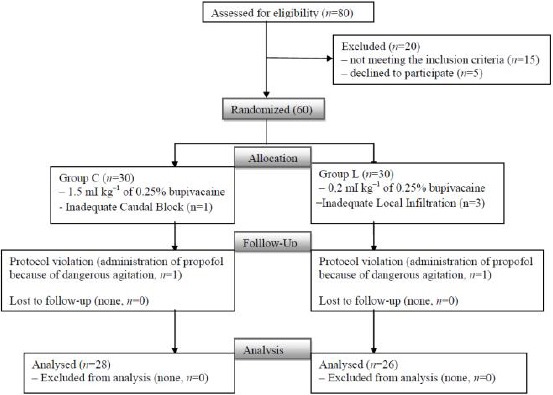
*Consort diagram. C: Caudal Group, L: Local Infiltration Group*.

In this trial 80 patients were choose eligible for examination but for several reasons they were excluded (Fig. 1). A total of 60 subjects were enrolled in the study and six in total were excluded. Four subjects (one in Group C and three in Group L) were with inadequate caudal block/local infiltration and required additional analgesia in operation theatre.

Two subjects (one in Group C and one in Group L) were excluded because attending anesthesiologists administered propofol to treat agitation that could not be controlled by fentanyl administration. Therefore, these six subjects were all excluded from the study.

The two groups did not differ in terms of patient characteristic data and surgical profiles as well as hemodynamic changes between groups (Table 1, 2).

**Table 1:** Patient demographic data and other details (mean ± SD)

	Group Caudal	Group Local
	(n = 28)	(n = 26)
Gender (M/F)	23/5	21/5
Age (years)	3.6 ± 1.8	3.4 ± 1.9
Weight (kg)	14.6 ±4.4	16.5 ± 4.6
Duration of Anesthesia (min)	52.7 ± 9.0	53.5 ± 8.1
Duration of surgery (min)	34.9 ± 8.2	36.8 ± 8.1
Fluids (ml)	44.6 ±11.4	49 ± 10.9

**Table 2:** Hemodynamic changes between groups (mean ± SD)

	SBP		DBP		HP		SaO_2_	
Times points	Local (n = 26)	Caudal (n = 28)	Local (n = 26)	Caudal (n = 28)	Local (n = 26)	Caudal (n = 28)	Local (n = 26)	Caudal (n = 28)
T0	102 ± 9.3	103.4 ± 8.3	53.1 ± 7.2	55.6 ± 4.7	138.9 ± 31.5	149.3 ± 14.3	98.3 ± 0.6	98.3 ± 0.7
T1	95 ± 9.5	94.9 ± 8.3	49.6 ± 5.9	49.7 ± 4.4	114.3 ± 22.7	118.3 ± 13.9	99.3 ± 0.4	99.5 ± 0.5
T2	95.2 ± 9.1	92.7 ± 8.3	50.1 ± 6.2	48.3 ± 5.5	114.3 ± 15.6	117.8 ± 14.0	99.4 ± 0.4	99.6 ± 0.4
T3	93.7 ± 8.9	91.6 ± 8.8	47.3 ± 6.3	47.3 ± 6.4	111.8 ± 14.6	113.1 ± 14.1	99.4 ± 0.5	99.5 ± 0.4
T4	98 ± 9.7	94.2 ± 9.2	52.2 ± 6.1	49.4 ± 7.1	119.5 ± 18.8	122.4 ± 14.3	98.8 ± 0.4	99.3 ± 0.4

To – Baseline value; T1 – After induction in anesthesia; T2 – incision; T3 – surgery; T4- end of operation; HR- Heart rate; SBP – Systolic blood pressure; DBP – Diastolic blood pressure; SaO_2_%–Peripheral oxygen saturation.

The incidence of rescue fentanyl in the PACU and acetaminophen on ward was significantly lower in children who received caudal block compared to those who received local wound infiltration (Table 4). Two of 28 in the caudal group and nine of 26 in the local group received fentanyl rescue analgesia in PACU. Only one in 28 of the caudal group received acetaminophen on ward and neither one of 26 from the local group received acetaminophen on ward. The difference between groups for analgesic requirement is statistically significant p<0.05. Time to first analgesic administration was not statistically significant p=0.40.

**Table 3:** Postoperative analgesic profile in the groups

		CaudalGroup	Local Group
		(n = 28)	(n = 26)
No need of analgesic		25	17
N0 of subject with oral analgesic		3	9
One dose of analgesic		3	9
More than one dose of analgesic		0	0
for 24 hour			
Rescue fentanyl at PACU		2	9
Acetaminophen on WARD		1	0
	Min	15	15
Time to first analgesic	Max	840	90
requirement (min)	mean ± SD	325 ± 449	55 ± 30
	Vomiting	4	0
	Agitation	1	0
Complications	Urinary	1	0
	retention		
	Motor block	1	0
Analgesia	Opoids	2	9
	Acetaminophen	1	0

**Table 4:** Post anesthesia assessment in the groups in Post Anesthesia Care Unit

Assessments	Group Caudal (n = 28)	Group Local (n = 26)
CHEOPS	2.8 ± 1.6	4.2 ± 1.8
FLACCS	2.6 ± 1.4	3.8 ± 1.9
Post-operative sedation	0.3 ± 0.7	0.4 ± 0.4
Motor function	1.3 ± 0.5	0.0 ± 0.0

(CHEOPS = Children Hospital of Easter Ontario Pain Scale); (FLACCS= Face Legs Activity Cry Consolability scale)

Pain scores using CHEOPS and FLACC assessed at the PACU were significantly lower in the caudal group. As for sedation, it was similar in both groups and motor function was better in local group; all subjects had good motor function; in caudal group one of 28 was not able to move the legs and two were able to move their legs and 26 haven’t got motor dysfunction (Table 3).

There were significant differences in the incidence of adverse effects in caudal and local group including: vomiting (14.29% vs. 0%), delirium (3.5% vs. 0%), and in one subject from caudal group an urologist decided on urinary catheterization (3.5% vs. 0%). Vomiting was well controlled by a single dose of antiemetic. Delirium was controlled with single dose of midazolam. Adverse effects were not noted in the Local group. The time from when the patient entered the recovery room to when they met the discharge criteria did not differ in both groups; all subjects were discharged after 24 hours.

## Discussion

To our knowledge, this study is the first to examine the effects of local infiltration alone without ilio-inguinal and ilio-hypogastric nerve block on pain management after pediatric hernia repair surgery. We demonstrated that a single dose of local infiltrated bupivacaine 0.25% 0.2 ml/kg compared with caudal block does not reduce postoperative pain; and compared to caudal block, local wound infiltration is safe in terms of adverse events. In the review article of Martin Jöhe for regional anesthesia in neonates, infants and children, local infiltration can produce reliable analgesia and is widely used by pediatricians, surgeons and emergency physicians [1]. In the same article Jöhe mentions that ultrasound is not essential for performing a CB. On the contrary, it may make a simple procedure complicated and more prone to infection, however it can help in cases of suspected anomalies at palpation and also for teaching purposes [1]. In our study we tried to use the LWI anesthesia alone for hernia repair in children. We did not use ultrasound and failure rate was 3.3% for CB and 10% for LWI. There are studies comparing CB versus LWI but all of them include ilio-inguinal, ilio-hypogastic nerve block and local infiltration [11]. We identified 7 studies in total. Both interventions were performed after surgery in two studies [12-14]; however the other 4 studies performed caudal preoperatively and infiltration postoperatively [5, 13, 15, 16] and only one study performed both techniques preoperatively [17]. All included hernia surgeries only except for Lafferty and colleagues (only orchidopexy) [13]. All used bupivacaine in concentration of 0.25% for CB and 0.25%–0.5% for LWI. The volume ranged from 0.7 to 1.0 ml/kg (CB) and from 0.2 to 0.7 ml/kg (LWI). Only Conroy and colleagues used epinephrine along with bupivacaine [16]. Variations of the infiltration techniques involved infiltration of the wound site through the skin and infiltration of fascia or aponeurosis before closure. No study used image guidance. We performed both interventions preoperatively, CB with 1 ml/kg – 0.25% pure bupivacaine and LWI with 0.2 ml/kg – 0.25% also pure bupivacaine.

Since 1992 Ejlersen compared the efficiency of pre-incision and pos-incision wound infiltration with Lidocain 1% on the postoperative pain of adult patients with inguinal herniotomy. The demand for additional analgesics occurred earlier in those who received Lidocaine infiltration after incision. The pre-incisional infiltration group also had fewer patients requiring supplemental analgesic [18]. The findings of our study and the findings of Ejlersen suggest that the inhibition of peripheral sensitization may be of major importance in impeding the development of acute pain and explain why prevention is important in handling operative pain. There are no firmly established dosage schemes for either technique and each has reasonable alternatives. A 1 ml/kg dose of bupivacaine for CB is widely employed, simple and safe [19]. The best method of local anesthesia is unknown. Varieties of techniques have been used and include wound instillation, wound infiltration and local neural blockade of ilio-inguinal, ilio-hypogastric and genitor-femoral nerves [20]. The optimal concentration of bupivacaine is not known; also which combination of local blocks are optimal needs to be determined. For this study we chose a simple, yet effective technique, which we believe to be popular and clinically relevant.

There are several limitations to the present study. First we can’t close the eyes of the investigator who performed the intervention, which means that potential bias exists. There is also lack of long postoperative follow-up to evaluate whether there are other late-onset complications. Number of investigated subjects is low.

In conclusion, between children undergoing inguinal hernia repair, local wound infiltration insures safety and satisfactory analgesia for surgery. Compared to caudal block it is not overwhelming. Caudal block provides longer analgesia, however complications are rather common.
